# Epidemiological traits of the malaria-like parasite *Polychromophilus murinus* in the Daubenton’s bat *Myotis daubentonii*

**DOI:** 10.1186/s13071-014-0566-7

**Published:** 2014-12-09

**Authors:** Fardo Witsenburg, Franziska Schneider, Philippe Christe

**Affiliations:** Department of Ecology and Evolution, University of Lausanne, Biophore, CH-1015, Lausanne, Switzerland

**Keywords:** Polychromophilus, Myotis, Bats, Apicomplexa, Haemosporida, Susceptibility, Chiroptera

## Abstract

**Background:**

The great diversity of bat haemosporidians is being uncovered with the help of molecular tools. Yet most of these studies provide only snapshots in time of the parasites discovered. *Polychromophilus murinus*, a malaria-like blood parasite, specialised on temperate-zone bats is a species that is being ‘rediscovered’. This study describes the infection dynamics over time and between host sex and age classes.

**Methods:**

For three years we followed the members of three breeding colonies of *Myotis daubentonii* in Western Switzerland and screened them for the prevalence and parasitemia of *P. murinus* using both molecular tools and traditional microscopy. In order to identify more susceptible classes of hosts, we measured, sexed and aged all individuals. During one year, we additionally measured body temperature and haematocrit values.

**Results:**

Juvenile bats demonstrated much higher parasitemia than any other age class sampled, suggesting that first exposure to the parasite is very early in life during which infections are also at their most intense. Moreover, in subadults there was a clear negative correlation between body condition and intensity of infection, whereas a weak positive correlation was observed in adults. Neither body temperature, nor haematocrit, two proxies used for pathology, could be linked to intensities of infection.

**Conclusion:**

If both weaker condition and younger age are associated with higher infection intensity, then the highest selection pressure exerted by *P. murinus* should be at the juvenile stage. Confusion over the identities and nomenclature of malarial-like parasites requires that molecular barcodes are coupled to accurate morphological descriptions.

**Electronic supplementary material:**

The online version of this article (doi:10.1186/s13071-014-0566-7) contains supplementary material, which is available to authorized users.

## Background

Bats are the hosts to a large array of blood parasites (e.g. *Bartonella*, piroplasms, trypanosomes and microfillaria). In particular, bats are host to a unique collection of Haemosporida (Apicomplexa). Besides *Plasmodium* spp. and the rarer *Hepatocystis* spp., which infect several orders of Mammalia, bats host at least two unique genera not found outside the Chiroptera [1], *Nycteria* and *Polychromophilus*, plus two additional genera known from a single record (*Dionisia* [2] and *Biguetellia* [3]). The diversity of haemosporidian parasites of bats suggests, together with a recent phylogeny [4], that the Chiroptera might have been the original host of all mammalian Haemosporida.

Naturally, this has sparked renewed interest in bat haemosporidians, and *Polychromophilus* spp. have seen a particular rise in attention. The increasing ease and efficiency of molecular techniques has facilitated the detection of these parasites, enabling studies on its prevalence [5,6], phylogenetic origins [7] and the discovery of new species [8]. However its development in a host population both through time and between host classes and other ecological measures are lacking, keeping large parts of its biology still a mystery. The aim of the current study is to describe these parameters for a temperate-zone member of the *Polychromophilus* genus, *P. murinus*. Its type host is *Vespertilio murinus*, but an important reservoir species seems to be *Myotis daubentonii*, the Daubenton’s bat, a common bat species spread across the Palearctic [1,6,9].

Though its epidemiology is practically unknown, the life cycle of *P. murinus* is surprisingly well described, both in the host as well as the vector. Exoerythrocytic schizogony initially takes place in several organs, but finally develops inside the Kupffer cells of the liver [1,10]. The gametocytes are the only stages invading erythrocytes and these are taken up by the bat fly *Nycteribia kolenatii* (Nycteribiidae: Diptera). Here sexual reproduction takes place, after which an oocyst develops sporozoites on the gut wall [11].

The distribution of *P. murinus* has been documented in very few studies but since it has been observed in Italy, Switzerland and Great Britain [6,11,12] it is likely that *P. murinus* has established itself in populations of *M. daubentonii* across Europe. In contrast, we have little idea about the prevalence variation over time, as well as the development of infection over the summer season. The aim of the current study was therefore to determine these epidemiological parameters in *M. daubentonii* at a local site*.* Secondly, by observing the susceptibility of each host age and sex class, the most likely source of the epidemic can be identified, which provides clues how the infection is maintained at this site. We will use both molecular methods, for parasite detection, and microscopy, for visual confirmation and assessment of the intensity of infection.

By definition, a parasite should have a negative effect on the host’s fitness. The direct effects of an infection with a haemosporidian parasite can be very severe [1,13], but may depend heavily on both the parasite and host species [14]. The only attempt at studying the physiological effects of *P. murinus* on bats failed when the artificially inoculated heterospecific host appeared unsusceptible to infection [15]. The attempt to infect a human with *P. murinus* by injecting infected bat blood failed as well; though the test subject did develop a fever, this was probably caused by other pathogens introduced by the injection [1]. Our aim here is not to perform a clinical experiment, but to explore possible effects of *P. murinus* infection using two physiological characteristics that can be used as proxies for pathology: haematocrit for anaemia and body temperature for fever.

## Methods

### Sample collection

*Myotis daubentonii* were captured during the seasons of 2010, 2011 and 2012 on the University of Lausanne campus, near Lake Geneva in Switzerland. The bats originated from three breeding colonies located in the forest on campus and were caught using a harp trap positioned over the Sorge stream at dusk. While highly pregnant and lactating females were immediately released upon capture, all other bats were used for sampling. Each bat was ringed to prevent resampling. The age of the bat, either ‘adult’ or ‘subadult’ was determined by the presence of a dark spot on the lower lip, which fades after 1-2 years. Each bat’s forearm length (to the nearest 0.1 mm) and weight (to the nearest 0.1 gram) were measured. We used the residuals from an OLS regression of body mass on forearm length as a measure of body condition [16]. In 2012, the haematocrit value (see below) and body temperature were also measured. Captured bats were immediately removed from the trap and body temperature was measured by inserting a lubricated probe into the rectum (RET-3 animal rectal probe and BAT-12 microprobe thermometer, Physitemp, Clifton, USA).

Blood was obtained by puncturing the uropatagial vein with a 0.5 mm gauge needle (Neolus). Between 5 and 30 μL of blood were collected using either microvettes with EDTA (Sarstedt; seasons 2010/2011) or heparinized glass microcapillary tubes (Marcel Blanc & Cie; season 2012). Samples were stored at -20°C until molecular analysis. Haematocrit was measured by centrifuging the microcapillary tubes containing fresh blood for 7 minutes at 12,800 rpm. The haematocrit value was calculated by dividing the length of the red blood cell column by the total length (measured to the nearest 0.1 mm).

After blood sampling, haemostatic cotton was applied to the punctured vein until bleeding ceased. All bats were captured under the licenses #1317 and #1656, authorized by the Cantonal Veterinarian Service of Vaud, Switzerland.

### Blood parasite observation

From each blood sample, one drop of fresh blood was applied to a microscope object glass to make a thin smear. Slides were subsequently dried and immediately submerged in 100% methanol for fixation. Finally, a 5% Giemsa-stain was applied for one hour to stain the cells. Slides were inspected with a light microscope at 600× and 1000× magnification. *Polychromophilus murinus* gametocytes were identified following Garnham [1] and Landau *et al.* [17]. Of a subset of gametocytes we noted the sex, counted manually the number of pigment granules and measured the dimensions of cells and nuclei.

The relative abundance of *P. murinus* gametocytes in the blood was estimated by scoring the number of *P. murinus* gametocytes observed in each smear at 600× magnification for 15 minutes. This measure, from now on referred to as *parasitemia*, should give an idea of the intensity of infection.

### Molecular analysis

DNA was extracted using the Blood and Tissue spin column kit (Qiagen, CA), following the manufacturer’s tissue protocol, with an overnight digestion and eluted in 2 * 50 μL. To control for contamination, a negative control was always included during the extraction process. The infection status of the bat host was determined by detecting the presence of a 705 bp fragment of the mitochondrial cytochrome *b* gene (cyt*b*) of the parasite following a nested PCR protocol. Primers, reagent concentrations and thermal profiles can be found in Megali et al. [6]. Amplified fragments were run on a 1.5% agarose gel and visualised under UV light. For every eight samples tested, a negative control was included in the amplification protocol. Each sample was tested in two independent tests. Samples with ambiguous results were tested again in duplicate. Samples remaining ambiguous were discarded from the data set.

A previous study at this site had demonstrated that the local *M. daubentonii* bats were exclusively infected by *P. murinus* and that this parasite population consisted of seven, almost identical cyt*b* haplotypes [6]. The aim of our current study was not to describe the parasite molecularly. However, to confirm these previous findings, as well as our microscopic observations, we had a subset of the PCR positive samples purified and sequenced by a commercial agent (Microsynth, Switzerland) using both the forward and reverse primers of the nested reactions.

### Statistical analyses

Factors influencing *P. murinus* prevalence, as assessed by the nested PCR protocol, were identified by logistic regression. The original model included the variables *sex, age, body length* and *condition*, as well as *year* and *date*, the latter variable expressed as standardized days since April 1^st^. Interactions were included based on their biological relevance: *year x date*, *date x condition*, *sex x condition* and *age x condition*. Terms were removed by backward selection, based on AIC and non-significant residual deviances until a minimal adequate model was found.

Since the parasitemia data was zero-inflated and showed signs of overdispersion, a zero-inflated (i.e. ‘mixed’) negative binomial approach (ZINB) was implemented using the *pscl* package for R [18]. To identify factors that influence *P. murinus* parasitemia in wild *M. daubentonii* hosts, this model considers the overly abundant zeros to come from two different processes, i.e. ‘false zeros’, caused either by poor observations or individuals that did not encounter the parasite, and ‘true zeros’, resulting from the covariates being unfavorable for the parasite [19]. For both the false-zero portion of the model, which attempts to discriminate between the two types of zeros, and the count portion, the same host-related covariates were used as for the previously described logistic regression of infection status. However, the variable *date* was also included in its squared form after graphical inspection of the data. A backward selection procedure was adopted wherein a term was dropped from either the false-zero or count portion of the model until no further decrease in AICc was observed. Each progressive model was tested for a significant change in log likelihood compared to its predecessor [19].

Juveniles were collected only in September and October of 2012. These are fledged young-of-the-year, that can be recognized by the incomplete ossification of their finger joints. Because of their age, it is not recommended to capture them earlier in the season and they were therefore not included in the previously described analyses. A comparison between juveniles and the other two age classes was therefore done separately. An F-value was obtained by performing an ANOVA on parasitemia by age class, with *date* as a covariate. This F-value was compared to a null distribution obtained by randomizing *age* 999 times.

The effects of parasitemia on bat hematocrit values and body temperatures were tested by linear regression. To linearize the relationship, *parasitemia* was log(*x* + 0.5) transformed. As female mammals can have lower hematocrit levels [20] and higher body temperatures [21], sex was included as a cofactor in each analysis.

All statistical analyses were calculated using R ver. 3.0.1 [22].

## Results

**Figure 1:**
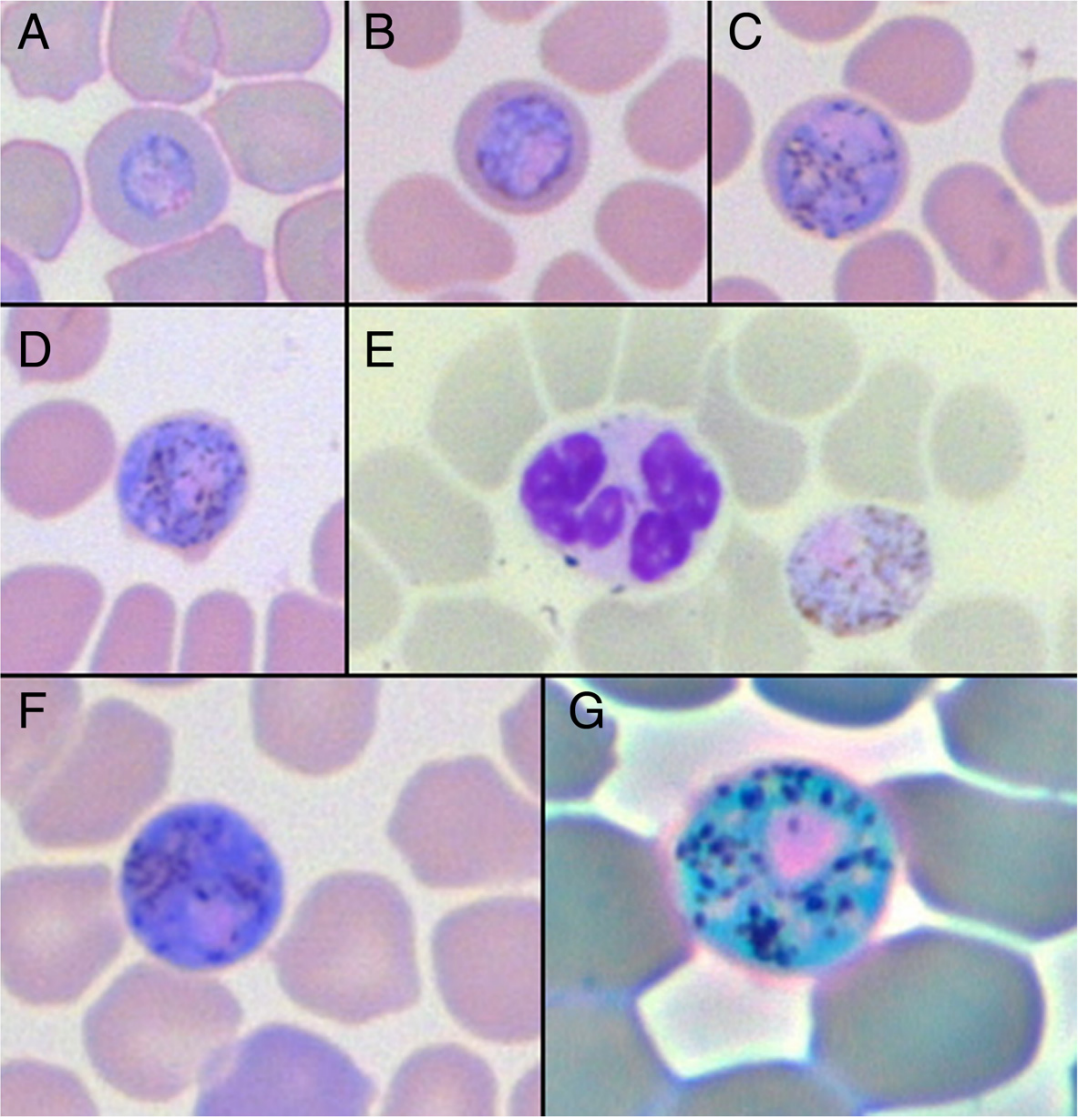
**Images of** ***Polychromophilus murinus*** **gametocytes in** ***Myotis daubentonii*** **erythrocytes. (A-D)** Different maturation stages of a gametocyte; **(E)** Mature male, microgametocyte with leucocyte; **(F)** Mature female, macrogametocyte; **(G)** Mature female, macrogametocyte, phase-contrast filter. Images A-F 600x magnification, image G 1000x magnification.

*P. murinus* gametocytes were detected in 58 blood smears (Figure 1). Gametocytes were large (Table 1), round to oval shaped, with a maximal diameter concurring with the described 6-8 μm [1]. Mature gametocytes would practically fill up the host cell, leaving mainly the edge of the host cell visible. This follows the description of Garnham [1], yet Landau *et al.* [17] mention that they do not completely fill up the host cell, but how much of the host lumen remains is not mentioned. The pigments are mouse-dropping shaped and of an irregular size, irregularly distributed across the cell; these clusters of pigments made counting them an ambiguous task and pigment numbers (Table 1) should therefore be taken with due caution.

**Table 1:** **Morphometric measurements taken of the gametocytes, in** μ**m**

	**Macrogametocyte**		**Microgametocyte**	
	**Range**	**Mean ± S.D.**	**Range**	**Mean ± S.D.**
**Cell length**	6.69 – 8.29	7.45 ± 0.38	7.29 – 8.64	8.01 ± 0.44
**Cell width**	5.50 – 7.84	6.97 ± 0.52	5.45 – 8.05	7.10 ± 0.72
**Nucleus length**	1.58 – 2.56	2.12 ± 0.23	3.08 – 5.95	4.66 ± 0.67
**Nucleus width**	1.34 – 2.10	1.7 ± 0.21	2.15 – 4.78	3.29 ± 0.63
**Number of pigment granules**	11- 29	20.30 ± 4.52	12 - 27	19.3 ± 5.54

The slightly smaller female macrogametocyte (n = 26) had a clearly defined compact nucleus, whereas the nucleus of the male microgametocyte (n = 16) was large and diffuse with ill-defined borders.

The female macro- and male microgametocytes are easily distinguished, with the macrogametocyte having a compact, round, well-bordered nucleus in a dense cytoplasm. In contrast, the often more oval-shaped microgametocyte has a much lighter coloured cytoplasm with a diffuse, ill-defined and randomly shaped nucleus, which is considered a clear characteristic of *P. murinus*. Due to its irregular shape, measuring the male nucleus width in a consistent way was challenging and the values should again be taken with caution (Table 1).

Of the 212 *M. daubentonii* tested, 157 (74.1%) were PCR positive for the cyt*b* fragment. Of the 186 individuals of which also the blood smear had been searched for parasites, 58 (31.1%) were positive. 80 individuals were negative by microscopic analysis but positive by PCR and 48 were negative according to both methods. All nine successfully sequenced cyt*b* fragments had a 100% identity with *P. murinus*, six were identical with haplotype 1 (Genbank Accession HM055583) and the three remaining with haplotype 2 (Genbank Accession HM055584; following [6]).

**Figure 2:**
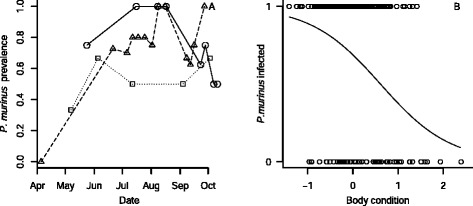
**Results from the logistic regression. (A)** Prevalence of *P. murinus* in local *M. daubentonii* through the season, separated by year. Circles and continuous line: 2010; triangles and dashed line: 2011; squares and dotted line: 2012. **(B)** The possibility of infection with *P. murinus* reduces with increased condition of the bat.

All required data for logistic regression analyses were available for 193 bats. Infection rates increased over the season, but only in 2010 a period of peak infection appeared, around July-August (Figure 2A); other years showing either an unsteady increase (2011) or a flat trend (2012). The final model of *P. murinus* prevalence contained both *date*, *year* and its interaction (Table 2). All other variables were retained as well, but not their interactions or *age* (Table S1 in Additional file 1). The goodness-of-fit test was not significant (Hosmer-Lemeshow, *X*^*2*^ = 8.558, *p* = 0.38), but visually many variables showed no pattern at all. Many intercorrelations existed between the predictors, which can make GLM solutions very sensitive to small variations in predictors [23]. To assess the robustness of our solution, we randomly split the data in two and three subsets, each subset containing respectively 50% or 33% of all observations, and used these to retest our model. Upon retesting, many variables disappeared from the model. Only date appeared as a reliable predictor and, with the exception of one model, body condition as well (Figure 2B; Table S3 in Additional file 1).

**Table 2:** **Estimates of the parameters and their significance for the logistic regression of prevalence**

**Parameter**	**Estimate**	**Standard error**	***X*** ^***2***^	***p***
*Year (2011)*	-0.783	0.545	-	-
*Year (2012)*	-1.996	0.650	-	-
*Date*	-0.247	0.417	-	-
*Sex (male)*	-1.061	0.471	5.32	0.021
*Condition*	-1.298	0.316	20.01	< 1*10^-5^
*Length*	-0.326	0.183	3.32	0.068
*Date x year (2011)*	1.045	0.506	6.36	0.041
*Date x year (2012)*	0.176	0.540	”	”

**Figure 3:**
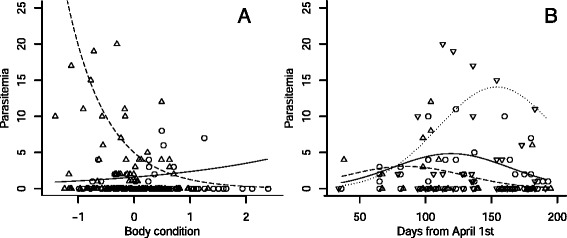
**Predictors of the intensity of infection as approximated by parasitemia. (A)** The relation between body condition and parasitemia differs between age classes. Circles and continuous trend line: adults; triangles and dashed trend line: subadults. **(B)** Parasitemia changes through the season and interacts with body condition. Continuous trend line and circles: bats with mean body condition; dashed trend line and up-facing triangles: bats in high body condition (>mean +0.5 s.d.); dotted trend lines and down-facing triangles: bats in low body condition (<mean - 0.5 s.d.).

**Figure 4:**
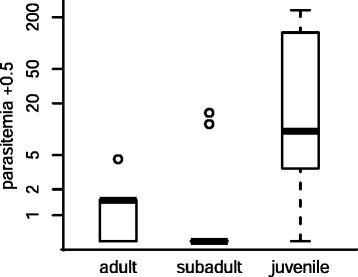
**The abundance of** ***P. murinus*** **gametocytes in the blood of different bat age classes**. Parasitemia of *M. daubentonii* caught in September and October 2012. Young of the year (juveniles) have significantly higher parasitemia than older age classes. The y-axis, the number of blood parasites observed, is on a log scale. For visualisation purposes, 0.5 is added to parasitemia.

Parasitemia data were collected from a total of 186 *M. daubentonii* for the analysis of blood parasite abundances. The minimal adequate ZINB model retained multiple terms in the count model, but none in the false-zero portion (Table 3, Table S2 in Additional file 1), though the difference between the last two models (with or without *age* in the false-zero part of the model) was only marginal (ΔAICc = 0.58; Table S2 in Additional file 1). The count portion of the ZINB model retained several variables. *Condition* and *age* had a significant interaction. In adults, parasitemia increased slightly with increasing body condition, whereas in subadults a strong negative relationship existed between *parasitemia* and *body condition* (Table 3, Figure 3A). In general, individuals in higher body condition had lower parasitemia, and the interaction with *date* indicated that peak parasitemia was reached sooner in bats in high body condition (Figure 3B). Though ranges overlapped, juveniles had higher median and maximum parasitemia by one and two orders of magnitude respectively (Figure 4, randomized F-test: *n* = 23, randomizations = 999, *p* = 0.014).

**Table 3:** **Estimates of the parasitemia statistical model parameters and their significance for the model**

**Parameter**	**Estimate**	**Standard error**	***X*** ^***2***^	***p***
**Count model μ**				
*Date* ^*2*^	-0.472	0.178	5.62	0.018
*Date*	0.189	0.189	-	-
*Condition*	0.396	0.525	-	-
*Age (subadult)*	1.144	0.381	-	-
*Date x condition*	-1.130	0.368	9.77	0.002
*Age x condition*	-1.778	0.679	6.31	0.012
**False zero model π**				
*None*	-	-	-	-

**Figure 5:**
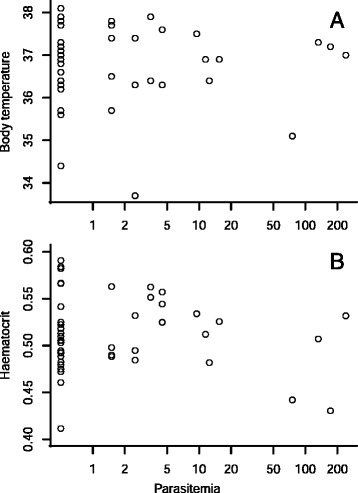
**The infection of** ***P. murinus*** **had no clear physiological effect on the bats. (A)** Body temperature in degrees Celsius; **(B)** Haematocrit, calculated as red blood cell volume fraction. The intensity of infection on the x-axis, expressed as parasitemia +0.5, is on a log scale.

Parasitemia of *P. murinus* had no effect on the body temperature of the bats when corrected for sex (multiple linear regression: *F*_*2,39*_ = 0.057; *p* = 0.943; Figure 5A). Hematocrit value did not appear to be influenced by the abundance of gametocytes either (multiple linear regression: *F*_*2,44*_ = 0.078; *p* = 0.463; Figure 5B).

## Discussion

*Polychromophilus murinus* reached its highest abundances in juvenile *M. daubentonii*, which have to carry the heaviest burden of infection. However, no direct physiological effect of infection was found. Furthermore, the zero-inflated model showed that across the whole season parasitemia was much higher in subadults than adults. This suggests that with age, bats are better able to cope with the infection.

Previous work has shown that the primary exposure to a haemosporidian parasite causes much higher parasitemia in hosts than any further encounters [1]. Our study demonstrated much higher parasite abundances in juveniles than other age classes, supporting this observation. Bat ectoparasites synchronise their reproduction with their hosts [24]. After female bats have given birth, the ectoparasites move in mass onto the pups [25], introducing blood parasites to the neonates at a very early stage of development. The primary exposure to the haemosporidian parasite causes extreme high levels of parasitemia, which in turn increases the probability of establishing an infection in the abundant newly emerged bat flies. As bat flies are long lived and can overwinter [15], this in itself might suffice to maintain the *P. murinus* infection.

The bat’s body condition was linked to the chance of being infected and it correlated with the progression of infection as well as the maximum intensity of the infection. Notably, the effect of host body condition on the parasite intensity depended on the age of the host, for which several non-mutually exclusive processes might be responsible. Subadults with larger fat storages could be better equipped to mount a costly immune response [26], though no direct relation was found in the Brazilian free-tailed bat *Tadarida brasiliensis* [27]. In *M. daubentonii* body condition increases with maturation [28]. The strong decline of parasitemia with condition seen in subadults might therefore actually represent maturing individuals which at the same time learn to cope with infection.

However, in adults, the relationship between condition and parasitemia is slightly positive. Heavier adults might be trading off mass against immunity, though it is unclear why this would only affect adults [26]. Like other bat ectoparasites [29], the vector *N. kolenatii* is attracted to hosts in higher body condition [30]; these bats might therefore be more often exposed to new infections, which causes a slight increase in parasitemia. It might also be a sampling artefact: perhaps for bats with similar levels of infection, only those in good condition can tolerate it enough to go foraging at night when we caught them. Lastly, the positive correlation might be caused by pregnant females, which are relatively heavy for their size and also immunosuppressed [25]. Though highly gravid females were never sampled, females in earlier stages of gestation are more difficult to recognize and might therefore be present in the data set.

It is worth noting, that in our statistical analyses we have considered a bat’s body condition as one of the causes predicting the likelihood of infection and its intensity. Yet, in contrast to date or age, the reverse is just as likely. Mounting an immune response requires energy which should reduce fat reserves [26]. Loss of body mass might therefore very well be a symptom of infection with *P. murinus*. Only experimental infections under controlled conditions could resolve this question of cause and effect.

Apart from possible weight loss, we found no other sign of pathology. Bats showed no signs of fever; however, whether or not fever is applied by heterotherms is debated [31]. The lack of anaemia on the other hand might be because of the biology of *P. murinus*. Unlike *Plasmodium* spp., *Polychromophilus* spp. have no asexual multiplication in the blood. The number of erythrocytes destroyed during an infection should therefore be much lower compared to other malaria species.

Over the three years we found an average infection rate of 74.1% which is the same rate as Megali *et al.* [6] found at the same site in 2009 using the same screening method. Prevalence was not stable throughout the season, but this pattern seemed different each year suggesting either random emergent fluctuations prevalence or the influence of (unmeasured) climatic variables.

Prevalence was much higher based on PCR than based on the microscopy results. It is well established that nested PCR is more sensitive than pure visual control, though the two methods can approach each other in efficacy [32]. The proportion of ‘false zeros’ determined by the ZINB model did not correspond to the number of bats tested negative by PCR. When all PCR-negative individuals were removed from the parasitemia analysis, zero-inflation was still an issue. The false zeroes were therefore not only caused by hosts that had not encountered the parasite, but also ‘bad observations’. In our case, the ZINB found no factor influencing the appearance of false zeroes, indicating that this rate of zero detection was unbiased across the categories of hosts. In contrast with this, the actual probability of being infected, as demonstrated by the logistic regression, depends mainly on time of season and the host’s condition.

The rise of molecular techniques in parasitology also inadvertently meant a decline of light microscopy [33], not only to describe new parasite species or lineages (but see: [34]), but also to determine parasite prevalence and abundance. Although PCR and qPCR are undeniably much more efficient approaches for analysing hundreds of blood samples at a time, some pitfalls are associated with the use of molecular techniques. Primers may be unsuitable for the parasite present [33] or only dead-end stages, injected by the vector, are detected [35], leading to the false conclusions that the host is uninfected or infected respectively. In this study we used PCR methods to scan for infections. Our main findings, however, stem from our results produced by microscopical investigation.

The currently available species descriptions for *P. murinus* are either not very exhaustive [1] or contradictory; Landau *et al.*, for example, would sometimes describe the *P. murinus* gametocytes as of the type *malariae* [2], yet in other publications as of the type *falciparum* [3], whereas Garnham describes it as of type *vivax* [1]. Here we tried to provide some quantitative measures with our morphological description of *P. murinus* to link the morphospecies to the available genetic sequences. With the current confusing species descriptions available, a proper redescription would be welcome. However, for a complete description it is essential to have access to the schizonts, which can be sampled by dissection. Such complete redescriptions coupled to sound genetic bar codes is needed to resolve the presently confused state of taxonomy among malarial-like parasites.

## Conclusion

Juvenile *M. daubentonii* clearly carried the highest *P. murinus* parasite loads. Furthermore, bat host condition was strongly correlated to the infection intensity. If both weaker condition and younger age are associated with a more intense infection, then the highest selection pressure exerted by *P. murinus* should be on the juvenile *M. daubentonii*. Weak young are pruned from the population and any surviving bats will have developed a tolerance against further infections with *P. murinus*. This could explain both the absence of any pathological symptoms in adults as well as the ability of the parasite to remain present in bat populations throughout the year.

## Additional file

Additional file 1:
**The file contains supplementary results of the statistical analyses.** **S1)** Table of the backward-selection procedure of the prevalence model. **S2)** Table of the backward-selection procedure of the parasitemia model. **S3)** Stability of the backwards selection procedure, by using subsets of the prevalence data set.
