# Supplementary material for: Epidemiological traits of the malaria-like parasite Polychromophilus murinus in the Daubenton’s bat Myotis daubentonii
Source: Parasit Vectors. 2014 Dec 9;7:566. doi: 10.1186/s13071-014-0566-7 (PMC4269171; doi:10.1186/s13071-014-0566-7)
Supplement: Additional file 1: — The file contains supplementary results of the statistical analyses. S1) Table of the backward-selection procedure of the prevalence model. S2) Table of the backward-selection procedure of the parasitemia model. S3) Stability of the backwards selection procedure, by using subsets of the prevalence data set. [file 13071_2014_566_MOESM1_ESM.pdf]

F. Witsenburg, F. Schneider & P. Christe, *The epidemiology of the malaria-like parasite* Polychromophilus murinus *in a population of Daubenton's bats*

Additional file 1: Supplementary Tables

Table S1. Backward selection of the logistic regression of prevalence. The most unsignificant terms were dropped until minimum AIC had been reached.

**Dropped variable**	**AIC**	**df**	**Residual deviance**	X^2	***p***
None (initial model)	214.71	179	186.71	-	-
age x condition	212.71	180	186.71	$4.96*10^{-06}$	0.9982
sex x age	210.77	181	186.77	0.064221	0.7999
sex x condition	209.15	182	187.15	0.38227	0.5364
age	208.10	183	188.10	0.94743	0.3304
date x condition	207.68	184	189.68	1.5781	0.209

Table S2. Backward selection of the parasitemia statistical model. Most unsignificant terms were dropped from the count- or false-zero model (respectively μ and π) until minimum AIC had been reached.

**Dropped variable**	**AICc**	**df**	**Loglikelihood**	X^2	***p***
None (Initial model)	537.03	29	-234.89	-	-
date x condition from π	534.30	28	-233.84	0.1065	0.7442
length from π	531.57	27	-233.88	0.0621	0.8032
length from μ	528.91	26	-233.92	0.0983	0.7539
$date^2$ *x year* from μ	525.51	24	-234.93	2.0146	0.3652
date from π	523.02	23	-235.02	0.1717	0.6786
sex x condition from π	521.15	22	-235.39	0.7532	0.3855
sex from π	519.86	21	-236.04	1.2984	0.2545
sex x condition from μ	519.68	20	-237.23	2.3795	0.1229
sex from μ	518.07	19	-237.69	0.9173	0.3382
year from μ	514.29	17	-238.28	1.1749	0.5557
year x date from π	511.39	15	-239.25	1.9413	0.3788
$date^2$ from π	509.52	14	-239.50	0.5067	0.4766
age x condition from π	509.01	13	-240.42	1.8424	0.1747
condition from π	506.81	12	-240.48	0.1124	0.7374
age from π	506.23	11	-241.34	1.7196	0.1897

Table S3. Stability of the backwards selection procedure, by using subsets of the prevalence data set. Variables retained in the model after backward selection are indicated by 'P' or 'N' depending on whether the estimate was positive or negative respectively. '-' indicates the absence of this variable in the considered model. Subsets with lowest AIC are in bold.

Subset	AIC	Year	Day	Year:day	Sex	Age	Condition	Length	Sex:age	Sex:condition	Condition:age	Condition:day
All	207.7	N	N	P	N	-	N	N	-	-	-	-
1st ½	114.9	-	P	-	-	-	N	-	-	-	-	-
**2nd ½**	**94.16**	**N**	**N**	**P**	**-**	**-**	**N**	**-**	**-**	**-**	**-**	**N**
**1st ⅓**	**64.56**	**-**	**P**	**-**	**-**	**-**	**N**	**-**	**-**	**-**	**-**	**N**
2nd ⅓	76.14	N	N	P	-	P	-	-	-	-	-	-
3rd ⅓	68.85	N	N	P	-	-	N	-	-	-	-	-
